# Modelling the Efficiency of Codon–tRNA Interactions Based on Codon Usage Bias

**DOI:** 10.1093/dnares/dsu017

**Published:** 2014-06-06

**Authors:** Renana Sabi, Tamir Tuller

**Affiliations:** 1 Department of Biomedical Engineering, Tel Aviv University, Tel Aviv, Israel; 2 The Sagol School of Neuroscience, Tel-Aviv University, Tel-Aviv, Israel

**Keywords:** codon usage bias, tRNA adaptation index, protein levels, wobble interactions, ribosome

## Abstract

The tRNA adaptation index (tAI) is a widely used measure of the efficiency by which a coding sequence is recognized by the intra-cellular tRNA pool. This index includes among others weights that represent wobble interactions between codons and tRNA molecules. Currently, these weights are based only on the gene expression in *Saccharomyces cerevisiae*. However, the efficiencies of the different codon–tRNA interactions are expected to vary among different organisms. In this study, we suggest a new approach for adjusting the tAI weights to any target model organism without the need for gene expression measurements. Our method is based on optimizing the correlation between the tAI and a measure of codon usage bias. Here, we show that in non-fungal the new tAI weights predict protein abundance significantly better than the traditional tAI weights. The unique tRNA–codon adaptation weights computed for 100 different organisms exhibit a significant correlation with evolutionary distance. The reported results demonstrate the usefulness of the new measure in future genomic studies.

## 1. Introduction

Allowed by the redundancy of the genetic code, coding regions exhibit non-uniform usage of synonymous codons. This deviation from uniform codon usage is termed codon usage bias (CUB) and is related among others to various aspects of gene translation (and more generally gene expression) and its efficiency;^1–10^ specifically, it was suggested that it regulates transcription and translation, but may also be related to recombination rate. Indeed, it is known for over 30 years that in most organisms the degree of CUB of genes correlates with their expression levels.^11–14^

Various approaches for quantifying the CUB of a gene have been suggested in the last decades: the effective number of codons, for instance, measures deviations from equal usage of synonymous codons,^13^ while other indices such as the frequency of optimal codons,^15^ the codon bias index,^11^ and the codon adaptation index (CAI)^16^ define a subset of ‘optimal’ codons and measure the frequency of these codons in the coding region of the gene.

The CUB indices have been employed in hundreds of previous studies. For example, it is known that in many organisms (e.g. *Escherichia coli*) the CAI exhibits a very high correlation with protein levels (similar to the one obtained between mRNA levels and protein levels^17^); thus, CAI has been frequently used as a proxy of expression levels (see, for example,^18–20^). In addition, it has been employed in a vast number of key studies in the recent years.^18,19,21,22^

One disadvantage of measures that are based on a set of reference genes^11,16,23^ is the fact that in the case of organisms with poor genomic data and without large scale cellular measurements, creating a meaningful reference set is challenging. Another disadvantage of these measures is the fact that they cannot separate between the various possible causes of CUB in highly expressed genes: some of them may be related directly to the translation process (e.g. co-evolution and/or adaptation to the tRNA pool^8,24,25^) and others may not be related to translation (e.g. GC content and folding^9,26–28^).

In 2004, dos Reis *et al.*^8^ proposed the tRNA adaptation index (tAI), which aims to estimate only the adaptation of codons/genes to some aspects directly related to the elongation step occurs in the ribosome via the adaptation to the tRNA pool, wobble interactions, and properties of the ribosome. Specifically, the tAI considers the fact that different tRNA species can recognize a codon with different affinities.^2,8,29^ Thus, the tAI is different than CUB-based measurements mentioned above and provides important information related to translation that is not necessarily covered by CUB measures.

Indeed, measures of the adaptation of genes to the tRNA pool (such as the tAI) have extensively been used in the recent years for studying questions in diverse biomedical disciplines such as evolutionary biology, functional genomics, and systems biology (see, for example,^3,30–35^).

Let *n_i_* be the number of tRNA isoacceptors that recognize the *i*th codon, the absolute adaptiveness value of the *i*th codon is defined in the following equation:

(1) $$W_{i}\;=\;\sum_{\;j=1}^{n_{i}}(1-S_{ij})\cdot \text{tGC}{\text{N}}_{ij}$$

where tGCN*_ij_* is the gene copy number of the *j*th tRNA that recognizes the *i*th codon (a proxy of the tRNA levels^24,29,36^), and *S_ij_* is a selective constraint on the efficiency of the interaction between the *i*th codon and the *j*th tRNA, which is scored between 0 (perfect interaction) and 1 (no interaction);^8^ specifically, the *S_ij_* weights can be related to aspects of translation elongation (tRNA, wobble interactions, and properties of the ribosome), as these aspects are expected to affect the efficiency of the codon–anticodon interaction. *W_i_* values are calculated according to Crick's wobble rules for codon–anticodon pairing (Table 1). The codon relative adaptiveness value *w_i_* is obtained by dividing each *W_i_* with the maximum *W_i_* value over all codons.^8^ The tAI of a gene is defined as the geometric mean of the *w_i_* values of its codons.

**Table 1.** Crick's wobble rules for calculating *W_i_*

	Codon third position		Anticodon first position	*W_i_*
*i*	U	*j*	I	$(1-s_{U:I})\text{tGC}{\text{N}}_{i,j}+(1-s_{U:G})\text{tGC}{\text{N}}_{i,j+1}$
*i* + 1	C	*j* + 1	G	$(1-s_{C:G})\text{tGC}{\text{N}}_{i+1,j+1}+(1-s_{C:I})\text{tGC}{\text{N}}_{i+1,j}$
*i* + 2	A	*j* + 2	U	$(1-s_{A:U})\text{tGC}{\text{N}}_{i+2,j+2}+(1-s_{A:I})\text{tGC}{\text{N}}_{i+2,j}$
*i* + 3	G	*j* + 3	C	$(1-s_{G:C})\text{tGC}{\text{N}}_{i+3,j}+(1-s_{G:U})\text{tGC}{\text{N}}_{i+2,j+2}$

The *W_i_* values are calculated based on Equation (1). The 64 codons are clustered in the genetic code into 16 groups, each one consists of four codons. The four codons in each group differ only in their third position (the wobble position). The formulas for calculating the *W_i_* values for each of the four codons in the group are given in the table. *i* denotes the index of the codon in the quartet which ends with U, *i* + *1*, *i* + *2*, and *i* + *3* denote the three other codons which end with bases C, A, and G, respectively. *j* denotes the index of the tRNA whose anticodon starts with I; all base pairing between the *i*th codon and the *j*th anticodon are WC. *j* + 1, *j* + 2, and *j* + 3 denote the three other tRNAs whose anticodons start with bases G, U, and C, respectively. tGCN*_ij_* represents the tRNA gene copy number corresponding to the interaction between the *i*th codon and the *j*th tRNA. For each codon, *W_i_* sums over all tRNAs that can pair with the codon. For example, the GCU codon which ends with U can either pair with anticodons that start with I (IGC) and generate a standard WC base pairing, or pair with anticodons that start with G (GGC) and generate a wobble base pairing.

In 1966, Crick^37^ suggested that in some cases wobble pairing may occur in the third base of the codon. According to Crick, the pairing at the third codon position has to obey chemical constrains; thus, some of the optional parings will not occur. For example, the unlikely pairing of guanine–adenine is due to a side group of guanine, which cannot make one of its bonds. In addition to the four standard nucleotides, modified nucleosides often occupy the wobble position of the anticodon (usually position 34 of the tRNA). In fact, the wobble position is the most frequently modified nucleoside in tRNA.^38,39^ Inosine, for example, is a common modification of adenine that occurs in the wobble position of many tRNA species.^39–43^

Out of the eight *S_ij_* weights, four are related to Watson–Crick (WC) interactions and the others are related to wobble interactions. In prokaryotic genomes, tRNA^IIe^ has a unique wobble position nucleoside [lysidine (L) in bacteria and agmatidine (agm) in archaea], which recognizes the AUA codon;^44^ thus, the prokaryotic *S_ij_* set contains one additional wobble *S_ij_* weight. The different possible pairs at the wobble position and the current tAI weights of the corresponding interactions are summarized in Table 2.

**Table 2.** The different base-pairings

*j*	*i*					
	I	G	U	C	A	L
I	—	—	*0*	**0.28**	**0.9999**	—
G	—	—	**0.41**	*0*	—	—
U	—	**0.68**	—	—	*0*	—
C	—	*0*	—	—	—	—
A	—	—	—	—	—	—
L	—	—	—	—	**0.89**	—

*S_ij_*-*values* are given to the pairing between the first position of the *j*th anticodon (tRNA) and the third position of the *i*th codon. *S_ij_*-values of WC base pairs are shown in italics, wobble values are shown in bold. Interactions which are not included in the calculation of the tAI are marked with hyphens. Lysidine (L) is a bacterial RNA modification of the DNA nucleotide cytidine (c).^44,45^

In the tAI, WC *S_ij_* weights are fixed to zero (perfect interactions) under the assumption of no constraint on these interactions. The wobble interaction weights are inferred by optimizing the correlation between gene expression levels (mRNA levels) and their corresponding tAI in *Saccharomyces cerevisiae*;^8,46^ the rationale behind this optimization is based on the following relations (which hold in many organisms): (i) there is correlation between mRNA levels and protein levels; (ii) there is correlation between translation rate and protein levels; and (iii) highly translated genes are under selection to include codons with higher adaptation to the tRNA pool.

The possibility of having different wobble interaction weights across different genomes has not yet been comprehensively studied. Here, we develop a novel generic approach for species-specific estimation of the tAI *S_ij_* weights without the need of gene expression measurements; for convenience, we name the new measure: species-specific tAI (stAI). This measure includes different *S_ij_* weights for each organism. We show that the correlation between protein levels and stAI is higher than that between protein levels and tAI.

Based on our approach, we infer the wobble *S_ij_* weights for a wide variety of organisms from the three domains of life, in order to examine the conjecture that organisms from different domains have significantly different *S_ij_* weights and to understand these differences.

## 2. Materials and methods

### 2.1. Computing the S_ij_ weights of the stAI without the need of gene expression measurements

The tAI weights are based on optimizing the correlation between tAI (Equation 1) and expression levels in *S. cerevisiae* and *E. coli.*^8^ However, large scale measurement of mRNA levels and specifically protein abundance (PA) are not available for most of the organisms with sequenced genomes.

To solve this problem we develop an approach that is based on the assumption that highly expressed genes should have both higher adaptation to the tRNA pool (i.e. higher tAI) and higher CUB (i.e. less uniform distribution of codons).^8^ Thus, there should be a significant correlation between CUB and tAI. Based on this assumption, we find the *S_ij_*-values that optimize the correlation between CUB and stAI. Note that the optimized correlation is at the level of genes while for each gene both measures are based on its codons content. Below we provide additional details about our approach including the CUB measure that we use.

### 2.2. Relative codon bias

In order to infer the *S_ij_* weights without the need of expression levels, we used a measure of CUB, which is based solely on the coding sequence. The strength of relative codon bias (RCBS) proposed by Roymondal *et al.*^47^ is an example of an index that is based only on the sequence. The RCBS of codon *xyz* is expressed as:

(2) $$d_{xyz\;}=\;\;\frac{\;f(x,y,z)\;-\;\;f_{1}(x)\cdot \;f_{2}(y)\cdot \;f_{3}(z)}{\;f_{1}(x)\cdot \;f_{2}(y)\cdot \;f_{3}(z)}$$

where *f*(*x*, *y*, *z*) is the observed frequency of codon *xyz* (where *x*, *y*, *z* denote the first/second/third nucleotides, respectively, of the codon) and *f*_1_(*x*), *f*_2_(*y*), and *f*_3_(*z*) are the observed frequencies of bases *x*, *y*, and *z* at, respectively, positions 1, 2, and 3 of the codon. These frequencies are computed for each gene separately. The RCBS of a gene of length *L*, in codons, is calculated as:

(3) $$\text{RCBS}\;=\;{\left(\prod_{i=1}^{L}\left(1+{d^{i}}_{xyz}\right)\right)}^{1/L}-1$$

RCBS takes into account base compositional bias, to get a more reliable measure of highly favoured codon frequency while controlling for other features of the coding sequence such as GC content bias.

According to Equation (2), rare codons will be given lower *d_xyz_* (i.e. a value close to −1) while a very frequent codon will be given a higher *d_xyz_* value (e.g. it can be 1). Thus, very rare codons decrease the final RCBS score of the gene and very frequent ones increase its final RCBS score (see Equation 3). However, we believe that (almost by definition) genes with very high CUB should include both very frequent codons and very rare codons. For example, if a hypothetical amino acid A has two codons, one is ‘optimal’, and the second is ‘not optimal’, we expect a very highly expressed codon usage biased gene to have a very high *d_xyz_* score for the first one and a very low *d_xyz_* score for the second one. But, we wish that both cases/codons will contribute to the same direction and increase the RCBS score.

Thus, we employ a modified version of the RCBS, which we term here directional codon bias score (DCBS), as in this measure, both positive and negative codon usage biases contribute (in the same direction) to the total CUB of the gene. We define the directional codon bias (DCB) of a codon triplet *xyz* as:

(4) $$d_{xyz\;}=\;\;\max\left(\frac{\;f(x,y,z)\;}{\;f_{1}(x)\cdot \;f_{2}(y)\cdot \;f_{3}(z)},\frac{\;f_{1}(x)\cdot \;f_{2}(y)\cdot \;f_{3}(z)}{\;f(x,y,z)}\right)$$

The DCBS of a gene of length *L*, in codons, is the following mean (see example in Supplementary data):

(5) $$\text{DCBS}\;=\;\frac{\sum_{i=1}^{L}d_{xyz_{}}}{L}$$

As we later demonstrate, in our framework the DCBS gives better results than the RCBS.

Finally, it is important to emphasize the fact that both RCBS and DCBS control for mutation bias. Specifically, when we compute the DCBS (see above), the frequency of each codon [f(x,y,z)] is normalized by the expected frequency under the assumption that the different nucleotides are independent [$\;f_{1}(x)\cdot \;f_{2}(y)\cdot \;f_{3}(z)$]; the later represents among others the mutation bias. The measure that we use is based on the frequency of the codon normalized by the expected frequency according to the mutation bias, and thus control for mutational bias (see also Supplementary data regarding the way our approach controls for possible factors affecting CUB).

### 2.3. Inferring the parameters of the stAI

The stAI inferred here is based on the same equations of the tAI with an organism-specific *S_ij_-*values' set (Equation 1), which is based on a measure of CUB. For every genome used in this study, the unique *S_ij_* set was inferred by optimizing the non-parametric (Spearman) correlation between DCBS (Equations 4 and 5) and stAI (Equation 1). To avoid convergence to local maxima point, we used various starting points. Specifically, we included in the set of starting points the original weights of the tAI^8^ and also the two extreme values of these weights (all zeros and all ones)*.* In order to choose a set of starting points, we halved the allowed region of the $S_{ij}$ values (i.e. the region: *S_ij_* between 0 and 0.5, and the region: *S_ij_* between 0.5 and 1) and considered all combinations for sampling values from these two regions (2^4^ possibilities for the four eukaryotic wobble *S_ij_* and 2^5^ for the five prokaryotic wobble *S_ij_*); thus, organisms from the same domain shared the same set of starting points. For each specific starting point, we used a hill climbing search method to iteratively optimize the *S_ij_* weights using a variable step size (starting with an initial step size of 0.3 and finishing with step size of 0.001). At each step size, when a new optimum was not found, the step size was decreased by a factor of 1.35. Iteration of the hill climbing included a random choice of *S_ij_* elements to change and a direction (i.e. increasing and decreasing) that increases the correlation between stAI and DCBS. The final chosen set of *S_ij_* was the one exhibited the maximum correlation between the stAI and DCBS. In order to determine whether the chosen set of starting points constituted a sufficient sample of the search space for the algorithm convergence, we added 100 more random starting points. The additional points provided no significant change in the final correlation between stAI and DCBS.

### 2.4. Comparison of the hill climbing method to Nedler–Mead search method

The Nedler–Mead (NM) optimization^48^ is the search method used to infer the *S_ij_*-values of the original tAI*.*^8^ When considering similar set of initiation points, our heuristic search outperformed the NM in finding the maxima of the objective function (i.e. the correlation between stAI and DCBS) in six of the eight model organisms (and was quite similar in the other two). We do not claim that hill climbing is better than NM; however, in the case of the specific problem analysed here (where the hill climbing explores the search space very well), and when considering the Matlab implementation of NM, the hill climbing was a bit better.

### 2.5. The analysed organisms

Our analysis included 100 different organisms (archaea, bacteria, and eukarya), in which CUB was correlated with the amount of adaptation to the tRNA pool. The correlation between stAI and DCBS/RCBS determined whether or not a tested genome would participate in the analysis. We excluded organisms in which an insignificant positive correlation or a significant negative correlation was observed; in such organisms, the assumptions that connect stAI to CUB do not hold and thus our method cannot be implemented. A detailed list of the excluded organisms is provided in Supplementary Table S1.

### 2.6. Generating randomized genes sequences

Random sequences were generated according to the real genomic codon distribution. For each of the 100 genomes studied in this work, 20 randomizations were performed by randomly drawing codons from the calculated genomic distribution and maintaining the protein content of the original genome.

### 2.7. Genomic sequences

In addition to the model organisms, which were chosen due to their available proteins measurements, we selected the genomes according to the list from ref.^49^, while trying to build relatively balanced group size wise (thus since bacteria was significantly larger than other groups in the list, we only included organisms of the three major phylums provided there: *Cyanobacteria*, *Alphprobacteria*, *Gamma-probacteria*).

Coding sequences of all 100 species were retrieved from the NCBI (ftp://ftp.ncbi.nih.gov/genomes/). Genomic tRNA copy numbers of all species except *Aspergillus nidulans*, *Debaryomyces hanasenii*, and *Candida albicans* were obtained from the Genomic tRNA Database (http://gtrnadb.ucsc.edu/). For *A. nidulans*, *D. hanasenii*, and *C. albicans*, we used the tRNA copy number as reported in ref.^30^. A detailed list of all organisms analysed here is provided in Supplementary Table S2.

### 2.8. Protein abundance

Large scale protein abundance (PA) measurements of *S. cerevisiae*, *E. coli*, *Arabidopsis thaliana*, *Shigella dysentariae*, *Caenorhabditis elegans*, *Drosophila melanogaster*, and *Leptospira interrogans* were retrieved from paxdB (http://pax-db.org/#!home). For *S. cerevisiae*, *E. coli*, *S. dysentariae*, and *L. interrogans*, a few datasets were provided. In this case, a weighted average between the different PA values was taken (i.e. we averaged the datasets after normalizing each of them such that they have identical average)*. Schizosaccharomyces pombe* expression levels were obtained from ref.^50^. The protein levels of some of the multiple cellular organisms were based on analysis of multiple tissues (*A. thaliana*, *D. melanogaster*, and *C. elegans*) (see details in http://pax-db.org/#!home). Specifically, we analysed all protein levels data that were available in paxdB (http://pax-db.org/#!home) on 2012. Note that in mammals it has been shown that the tRNA levels in various tissues tend to be correlative (the ranking of the tRNA genes abundance remains similar while the average value might change^51^); this is probably the case in many other organisms.

### 2.9. Permutation test for comparing two S_ij_ means

An empirical *P*-value was computed to test the null hypothesis that the means of two *S_ij_* distributions do not significantly differ between two groups of organisms; let *n* and *m* denote the number of organisms in the two groups, respectively. For each *S_ij_* component of the weights vector, we performed the following steps: first, we defined the normalized distance between the *S_ij_* means in the two groups of organisms as the absolute difference between the means divided by the sum of the two corresponding standard deviations (SDs). Secondly, we permute the *S_ij_* elements of the two groups by randomly drawing *n* values as the first group and *m* (non-overlapping) values as the second group. The random permutations were performed 100 times, each time the distance between the two random groups was computed. Finally, the *P*-value was defined as the number of times the random distance was higher or equal to the original distance divided by 100.

### 2.10. Spearman correlation as a measure to guide the optimization

The main advantage of this measure is the fact that it is a non-parametric measure that captures any monotonic relationship between CUB and stAI. Since this measure has been successfully employed in many papers in the field in this context,^18,52,53^ we decided to use it also here.

### 2.11. The general rational related to evaluating the stAI and demonstrating that stAI outperforms tAI

In this section, we would like to explain and emphasize the rational related to the analyses reported in this study. First, as mentioned in the section Introduction, CUB measurements such as the CAI quantify different gene expression aspects than the tAI. Here, we aim at improving the tAI (and not the CUB indices such as the CAI) and thus, our major baseline for stAI evaluations is the tAI (and not the CUB indices such as the CAI). Secondly, we use the correlations with PA as an indirect way to evaluate the stAI: we expect that genes with higher translation efficiency will have higher PA; we also expect that a better measure related to the adaptation to the tRNA pool will have higher correlation with translation efficiency; thus, we expect that a better measure related to the adaptation to the tRNA pool will have higher correlation with PA. It is clear that there can be CUB-based measurements with higher correlation with PA than stAI (see, for example,^54^)—however, as mentioned, the aim of this study is not to infer PA predictor but to improve the inference of the tAI parameters.

## 3. Results

### 3.1. The correlation between the CUB and tRNA pool varies among different organisms

A correlation between CUB and stAI is expected; however, the strength of this correlation among different organisms can teach us about the evolutionary forces shaping their genomes.

The correlations between stAI and DCBS obtained in the algorithm vary from a lowest value of 0.1136 (for the archaea *Halomicrobium mukohataei*) to a highest correlation of 0.7626 (for the fungi *Yarrowia lipolitica*). The bottom 10% correlations were obtained in prokaryotic genomes (the four archaea: *H. mukohataei*, *Archaeoglobus fulgidus*, *Pyrobaculum aerophilum*, and *Metallosphaera sedula*; and the six bacteria: *Anabaena variabilis*, *Brucella suis*, *Gloeobacter violaceus*, *Prochlorococcus marinus* MIT9313, *Synechococcus elongates*, and *Trichodesmium erythraeum*); thus, in this organisms, selection for CUB is presumably either weak or/and not strongly related to translation elongation and the tRNA pool.

The top 10% of the correlations were obtained mainly in eukaryotic genomes (the eight fungi: *C. albicans*, *C. glabrata*, *Eremothecium gossypii*, *Saccharomyces bayanus*, *S. mikatae*, *S. paradoxus*, *Cryptococcus neoformans*, and *Y. lipolitica*; and the two bacteria: *E. coli* and *Pasteurella multocida*); in these organisms, the selection for CUB is probably strongly related to the tRNA pool and translation elongations. All correlations are reported in Supplementary Table S3.

### 3.2. The stAI exhibits better PA predictions than the tAI in non-fungal organisms

**Figure 1.**
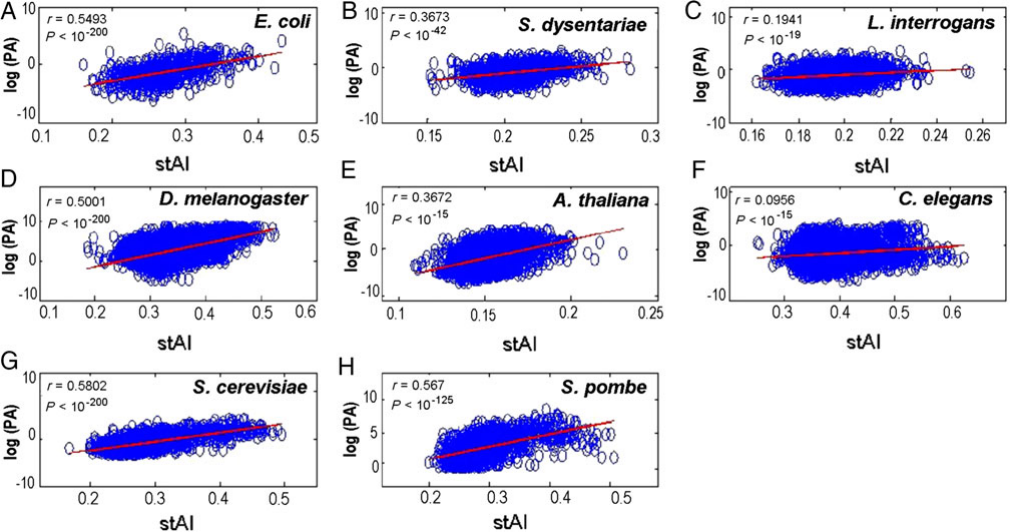
Dot plots of log(PA) vs. stAI and the corresponding Spearman rank correlations between stAI and PA. The correlations (and *P*-values) are calculated for the eight model organisms with PA measurements which include three bacteria (A–C), three non-fungal eukaryotes (D–F), and two fungi (G–H).

The correlations between stAI and PA are presented in Fig. 1. All eight models showed significant correlations. In six of the eight organisms, the correlation between stAI and PA was higher than that between tAI and PA. This result (Table 3) indicates that stAI outperforms the current tAI as a predictor of PA in all non-fungal organisms. For the two fungi used here (*S. cerevisiae* and *S. pombe*), the original tAI predicted PA better than the stAI*.* This result is not surprising since the *S_ij_*-values in the tAI were inferred based on the optimization of the correlation between tAI and *S. cerevisiae* mRNA expression levels^8^ (which strongly correlates with PA in *S. cerevisiae*; Spearman correlation of 0.74, *P*< 0.0001^55^); on the other hand, stAI is based on CUB, which is a less accurate measure of protein levels. However, for most of the sequenced genomes exist to date, expression levels are not available; thus, the stAI is valuable.

**Table 3.** Spearman rank correlation of the original tAI and the stAI with PA

	Number of genes	Number of proteins	*r* (tAI, PA)	*r* (stAI, PA)	Change (%)
Non-fungal					
* E. coli*	4,145	688	0.5032	0.5493	+8.39
* S. dysentariae*	4,501	1,266	0.3574	0.36757	+2.76
* L. interrogans*	3,667	2,114	0.0959	0.19408	+50.58
* A. thaliana*	28,163	8,478	0.3328	0.3762	+11.53
* C. elegans*	22,830	6,959	0.0919	0.0956	+3.87
* D. melanogaster*	10,926	6,510	0.4878	0.5001	+2.46
Fungi					
* S. cerevisiae*	5,869	2,666	0.6915	0.5802	−19.18
* S. pombe*	5,017	1,464	0.6554	0.56715	−15.58

The correlations between tAI and PA vs. the correlations between stAI and PA in eight model organisms with available PA data. The third column refers to the number of genes with available PA measurements in each organism.

We emphasize that although previous studies reported a significant positive correlation between CUB and expression levels in the model organisms studied here,^12,23,56,57^ it is not trivial that *Sij* optimization based on CUB improves the correlation with protein levels. Specifically, CUB is correlated with protein levels, but mRNA levels and protein levels in different organisms are also usually correlated;^52,58,59^ thus it is not clear that *S_ij_* optimized based on the CUB of the organism necessarily have higher correlation with protein levels than the *S_ij_* optimized based on mRNA levels of *S. cerevisiae*.

### 3.3. Robustness analysis demonstrates that in non-fungal organisms the stAI outperforms the tAI in terms of the correlation with PA

**Figure 2.**
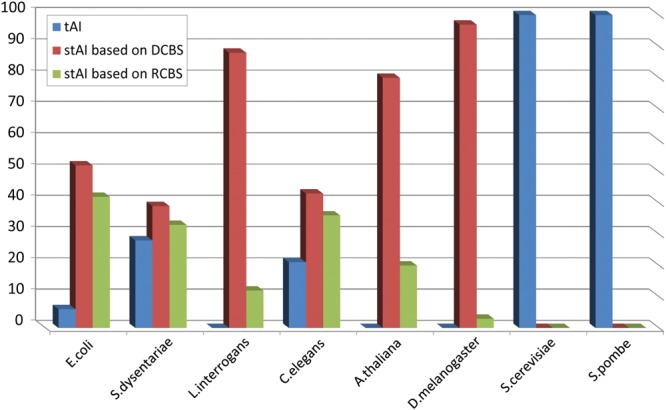
Comparison between stAI and the tAI. The middle bars representing the number of times (based on the jack-knifing analysis) the stAI outperformed the other versions of the tAI; as can be seen, stAI outperforms tAI in all non-fungal organisms.

In order to empirically estimate the organism-specific probability that stAI (which is based on DCBS) improves the correlation with PA, a jack-knifing approach was implemented. One round of it involved the implementation of the algorithm for calculating the stAI on a sample of random subset of 50% of the proteins. Finally, the correlation between stAI and PA was computed for the sample and was compared with the correlation of PA with two related indices: the original tAI and stAI that is based on RCBS (i.e. its *S_ij_* were inferred from RCBS and not from DCBS). This procedure was repeated 100 times where each time the index exhibited the highest correlation with PA was counted (Fig. 2).

As can be seen, the results demonstrate again that for non-fungal organisms, the species-specific inference of the *S_ij_* tends to predict PA better than the traditional tAI. The 100 *S_ij_* sets, their corresponding correlations between stAI and DCBS and the full taxonomy for each organism, are provided in Supplementary Table S3.

### 3.4. S_ij_ inferred based on CUB are similar to the S_ij_ inferred based on PA

In order to check whether the *S_ij_* that are inferred based on CUB (i.e. based on the DCBS) converge to similar values as those which are based on expression levels, we computed *S_ij_* sets by optimizing the correlation between stAI and PA for the model organisms with available PA measurements. This approach of using expression levels to optimize the tAI was employed in the study of ref.^8^. The Spearman rank correlation between the concatenated vectors of *S_ij_*-values (35 points) inferred based on the DCBS and the one inferred based on PA is 0.6902 (*P*-value <10^−5^; permutation *P*-value <0.001; 35 points). The Euclidean distance between the two vectors is also significantly lower than the one obtained by random permutation of the two vectors; specifically, when we performed 1,000 permutations of these values, all of them had higher Euclidean distance (*P*-value <0.001). The *S_ij_*-values that were obtained via correlation with DCBS and the ones obtained via correlation with PA are provided in Supplementary Table S4.

### 3.5. Considering all tRNA–codon pairing possibilities do not improve the performances of the stAI

There are possible cases of non-standard base pairing that currently are not included in the tAI wobble rules (U–U binding for instance). It is interesting to check whether introducing such additional rules to the model can improve its performances. Using Equation (1), we included in the set of *S_ij_* all missing pairing options (U:U, C:U, U:C, C:C, C:A, G:A, I:G, and G:G). An initial weight of 0.5 was given to all non-WC *S_ij_* (WC *S_ij_* are fixed to zero). Nevertheless, considering all possible pairings in the stAI weights calculation did not improve the correlation of the stAI with DCBS or with PA. The original approach (i.e. WC and wobble only) reached higher maxima values for seven of the eight models. In addition, for five of the eight models, better correlations with PA, were obtained for the original stAI.

### 3.6. Adding constraint on WC interactions do not improve the performances of the stAI

The first four *S_ij_* weights, which represent the constraint on WC interactions, are fixed to zero. Assuming that these interactions might not be perfect and thus allowing them to change during the optimization did not provide further improvement. The starting point for each model was the one exhibited the maximum correlation between stAI and DCBS in the original search. The original search, in which WC *S_ij_* are fixed to zero, reached higher maxima values for five of the eight models; in addition, in five of the eight models, the original search exhibited a better correlations with PA than the new search.

### 3.7. Distances between the inferred S_ij_-values correlate with evolutionary proximity

**Figure 3.**
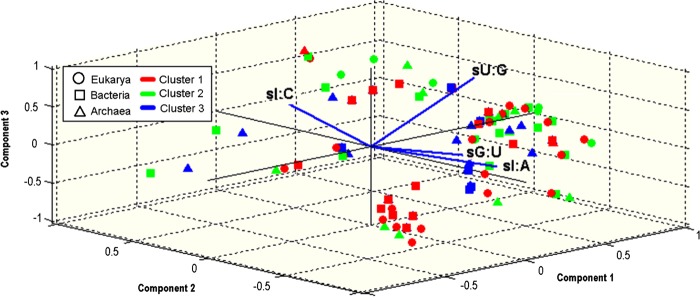
Principal component analysis (PCA) on the 100 different *S_ij_* sets demonstrates clustering of *S_ij_* according to evolutionary domains. The first three components of the *PCA* are presented. Each point in the figure represents one of the 100 analysed organisms; the shape of the point corresponds to the domain of the organism at the tree of life and the colour corresponds to the cluster the point was classified based on the *k-*means algorithm.

In the next step, we aimed at understanding if the organism-specific *S_ij_-*values reflect evolutionary proximity and if they are biologically meaningful. To this end, the inferred *S_ij_-*value sets of the 100 organisms were clustered into three groups using the *k-*means algorithm.^60^ We compared the clustering result to the clusters obtained by partitioning the organisms to the three domains of life. The clustering correctly classified 77% of the 26 eukarya, 45% of the 38 bacteria, and 67% of the 36 archaea (Fig. 3). In general, 61% of the total 100 organisms were classified into the correct domain.

Properly randomized genomes that were generated by maintaining the CUB of the genome and its protein content were used to empirically test the significance of this clustering (see section 2). None of the 20 randomizations outperformed the original clustering (with respect to total correct classifications, empirical *P*-value <0.05). This result demonstrates that with high probability the reported clustering cannot be obtained randomly even when considering randomized genomes with similar features to the original ones (global CUB and the same proteome), supporting the conjecture that the obtained *S_ij_-*value similarities correlate with the evolutionary distances and thus have biological meaning.

Finally, it is important to mention that there is co-evolution between CUB and tRNA levels (see, for example,^24,34,61,62^). Specifically, based on various theories, the CUB should co-evolve with the tRNA pool and the tRNA–codon interaction efficiencies to optimize the organism fitness; thus, CUB, tRNA levels, and tRNA–codon interactions cannot be separated.

### 3.8. Similarities among the inferred S_ij_-values of the analysed organisms

The mean efficiency of the different inferred codon–anticodon interactions over all the analysed organisms are summarized in Table 4. The results emphasize the similarities among the different organisms and domains.

**Table 4.** The mean inferred wobble *S_ij_*-values

	SG:U	SI:C	SI:A	SU:G	SL/agm:A
Eukarya	0.7861	0.4659	0.9075	0.6295	—
Bacteria	0.6294	0.4211	0.8773	0.698	0.7309
Archaea	0.3898	0.3774	0.5015	0.4363	0.6453
Mean	0.6	0.42	0.76	0.588	0.6881

The mean inferred wobble *S_ij_*-values strength for each domain of life and for the entire analysed dataset (last row).

As mentioned, *S_ij_*-values are between 0 and 1. Since these values represent a constraint on the codon–anticodon interactions, interactions with lower values are considered more efficient. For example, it can be seen from Table 4 that the inosine–cytosine interaction has the lowest mean value (*sI:C* = 0.42), while the wobble inosine–adenosine has the largest mean value (*sI:A* = 0.76). This suggests a good I:C interaction and an inefficient I:A interaction. These findings are supported by Murphy and Ramakrishnan.^63^ where it is stated that the decoding of adenosine-ending codons by inosine is inefficient. It is also mentioned that the inosine–cytosine interaction is very similar to the canonical G:C pair.

**Figure 4.**
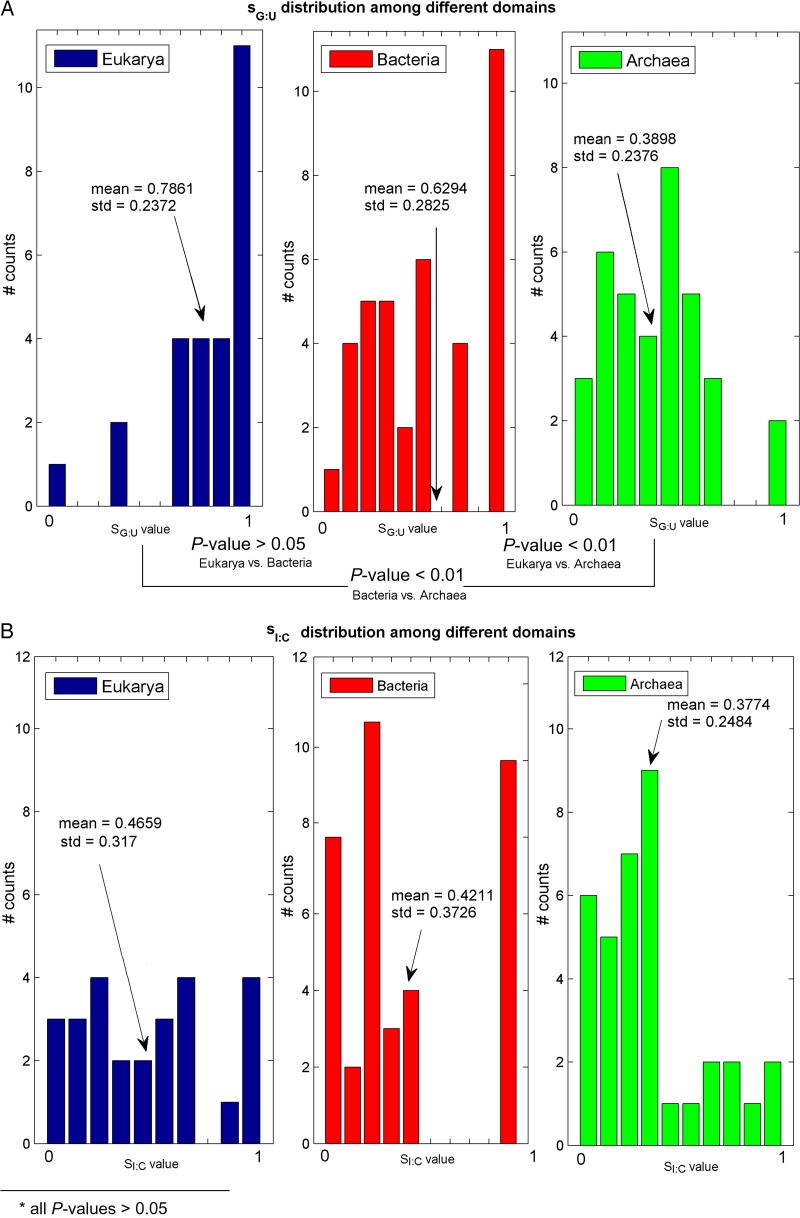

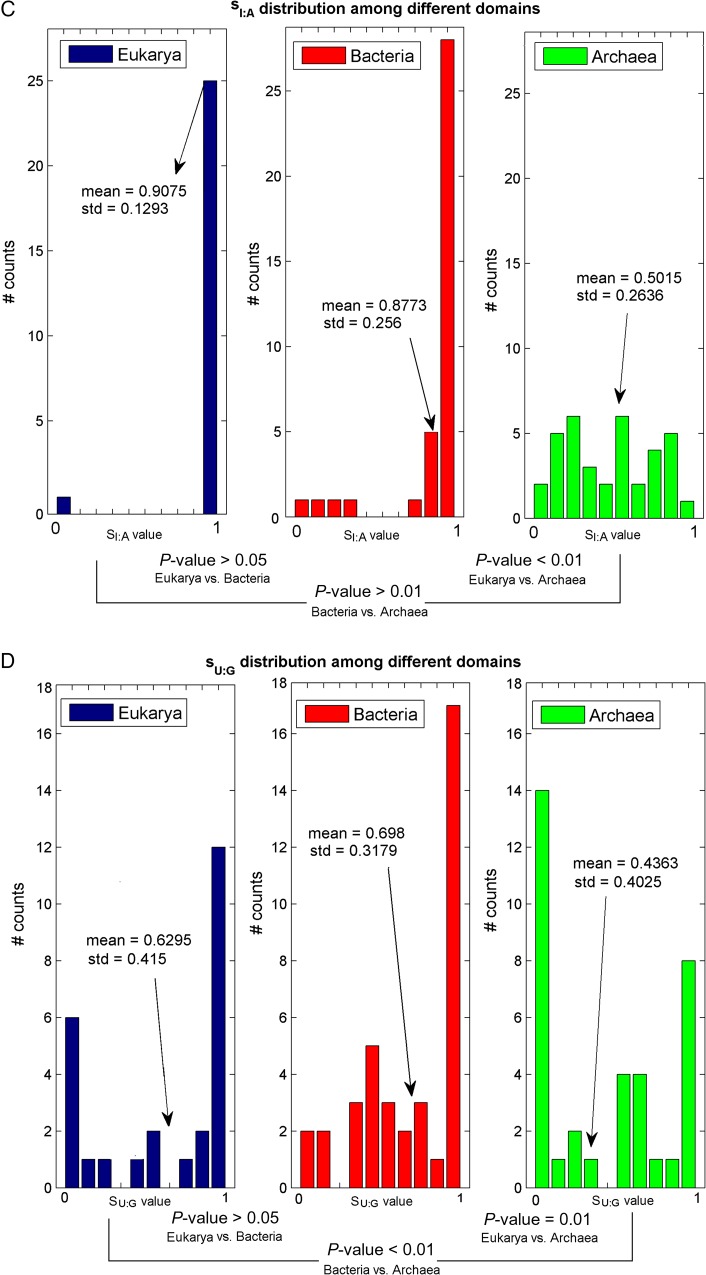

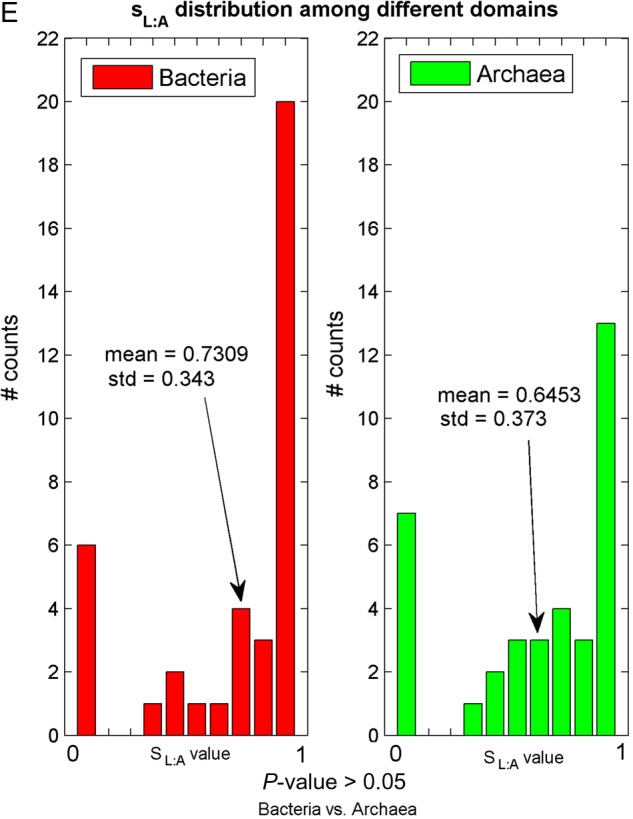
*S_ij_* distributions among different domains of life. Each figure contains three histograms representing the *S_ij_* in the different domains of life; the mean and SD of the *S_ij_*-values in each domain are also reported. The *P*-values corresponding to the comparison between every two *S_ij_* means appear in the bottom of the figure (see section 2 sub-section ‘Permutation test for comparing two *S_ij_* means’).

*SL:A* and *Sagm:A* have a similar distribution, and the corresponding *P*-value proves that the mean values are not significantly different (see Fig. 4E). Since agmatidine is in many ways similar to lysidine (see ref.^64^), it makes sense that their *S_ij_*-values are similar.

### 3.9. Differences among the inferred S_ij_-values of different groups of organisms

To test the hypothesis that the *S_ij_*-values of different organisms groups (i.e. different domains or different phylums within the same domain) have significantly different means, we computed an empirical permutation *P*-value (see section 2). The *S_ij_* distributions and their corresponding *P*-values are presented in Fig. 4.

**Figure 5.**
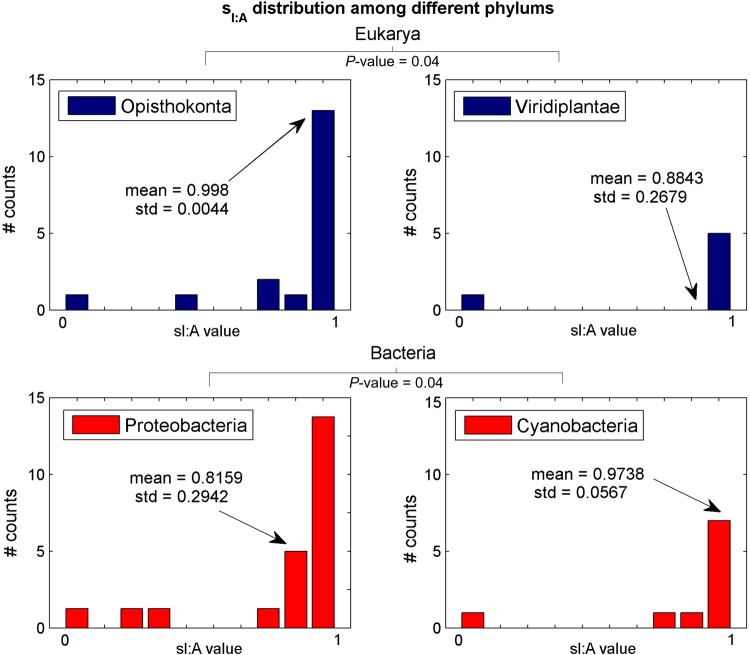
*sI:A* distribution within the major phylums of the eukaryotic and bacterial domains with a significant empirical *P*-value (see details in section 2).

As can be seen, the *sI:C* distribution is similar between the three domains (Fig. 4B); however, *sU:G*, *sI:A*, and *sG:U* tend to be significantly different among the three domains. An empirical *P*-value was used also for the comparison between the two major phylums within each domain. The only significant difference was obtained for the *sI:A* distribution of eukarya subgroups *Opisthokonta* vs. *Viridiplantae* and bacteria subgroups *Proteobacteria* vs. *Cyanobacteria* (see Fig. 5). All other insignificant *S_ij_* distributions among different phylums appear in Supplementary Fig. S5.

## 4. Discussion

In this study, we describe a new approach for inferring the efficiency of wobble interactions in the tAI without prior knowledge about the expression levels of the analysed organism. The approach is based on the fact that in most organisms highly expressed genes have higher CUB which is, at least partially, due to selection for improved adaptation of the codons to the tRNA pool of the organism. With our approach we infer the efficiency of wobble interactions via optimizing the component of the CUB that is due to adaptation to the tRNA pool (i.e. the correlation between these two measures: CUB and adaptation to the tRNA pool).

Thus, one limitation of our approach (and other CUB-based approaches) is the fact that it will not work in the case of organisms with no strong enough selection for both CUB and the adaptation to the tRNA pool in highly expressed genes; specifically, we assume that the evolutionary selection for this two phenomena tend to be stronger when the gene expression is higher.

In addition, we show that with our approach we are able to infer the efficiency of wobble interactions in non-fungal organisms better than the conventional approach (the tAI that does not optimize these values for each organism separately). In addition, we provide the estimations of these values for 100 organisms and show that they vary among different organism and correlate with evolutionary proximity. We report the similarities and differences among the inferred efficiencies of the analysed organisms.

PA measurements rather than mRNA level measurements are more appropriate for estimating the extent to which a coding sequence feature is related to translation efficiency. Thus, the improved correlation between stAI and PA exhibited for the non-fungal model organisms relatively to the correlation between tAI and PA demonstrates the advantages of our novel approach. Specifically, the improved correlation between stAI and PA indicates a strong association between translation efficiency (and thus PA), and the combined information the stAI provides which includes the co-adaptation of CUB to the tRNA pool, and the efficiency of the different wobble interactions.

Currently, there are less than a few dozen large scale measurements of protein levels, while there are >25,000 sequenced genomes. In addition, in the case of most of the organisms on earth, it is much easier to sequence their genomes, while it is usually impossible to culture them in order to measure their protein levels (see, for example,^65^). Our approach can improve the study of translation and evolution in such organisms, even if there are no available gene expression measurements.

The idea of different domains having different wobble *S_ij_-*values is supportive with the successful significant clustering reported in this study. The differences between the bacterial and eukaryotic ribosomes^66,67^ might provide a plausible explanation to this result as specific physical, chemical, and geometrical constraints are imposed on each tRNA–codon interaction. In the budding yeast, for example, the wobble inosine tRNA modification is essential for viability.^40^ This result is in line with a recent study^68^ that two kingdom-specific tRNA modifications are major contributors that separate archaeal, bacterial, and eukaryal genomes in terms of their tRNA gene composition. Specifically, with our approach, we were able to provide information about the interaction efficiencies that tend to vary among the different domains (*sU:G*, *sI:A*, and *sG:U*) and within some of the domains (*sI:A*); in addition, we show that the efficiencies of some of the interactions are conserved in all the domains (*sI:C*). Combining this information with additional information such as phylogenetic analysis, three dimensional conformations of the ribosome and tRNA molecules and knowledge related to tRNA modifications can provide a better understanding of the exact structure of the ribosome and tRNA molecules, their biochemical interactions, and their evolution.

We further verified that modelling non-conventional interactions between nucleotides does not significantly improve our model. Thus, our analysis supports the conjecture that, in the analysed organisms, wobble/WC interactions/parameters that appear in the original tAI measure should not be updated.

Finally, there has been a debate about the causality of the tRNA adaptation/protein level relations. Some previous studies suggested that increasing the adaptation to the tRNA pool has direct effect on translation rate and thus on protein levels.^34,69^ However, other studies have suggested that this relation is not causal: endogenous highly expressed genes have higher adaptation to the tRNA pool via reasons that are not directly related to the translation rate.^62,70^ For example, it has been suggested that the adaption of highly expressed genes to the tRNA pool improves the global ribosomal allocation among genes based on the fact that genes with higher adaptation to the tRNA pool consume less ribosomes;^9,70,71^ it was also suggested that evolution maintains a balance between codon frequency and the cellular levels of the tRNA genes such that the actual translation elongation speed is constant;^62^ highly expressed genes have higher adaptation to the tRNA pool since the effect of these genes on maintaining this balance is higher than in the case of lowly expressed genes.^62^ It is important to mention that the success of our approach is robust to the outcome of this debate. The fact that highly expressed genes have higher adaptation to the tRNA pool as reflected by the wobble interactions and the cellular tRNA levels is enough for the success of our approach, the exact biophysical/evolutionary mechanism does not matter.

## Supplementary Data

Supplementary data are available at www.dnaresearch.oxfordjournals.org.

## Funding

This study was supported in part by a fellowship from the Edmond J. Safra Center for Bioinformatics at Tel-Aviv University.
